# Supplementary material for: ClNAC100 Is a NAC Transcription Factor of Chinese Fir in Response to Phosphate Starvation
Source: Int J Mol Sci. 2023 Jun 22;24(13):10486. doi: 10.3390/ijms241310486 (PMC10341961; doi:10.3390/ijms241310486)
Supplement: Supplementary file 1 [file ijms-24-10486-s001.zip › CDS sequence and promoter sequence of ClNAC100.pdf]

ClNAC100 CDS sequence：

1245bp

ATGGAGGCCATGAATGGGAAGATGATGAATAGGATTGATGATGAGAATCTGCCCCCAG
GATTCAGGTTCCATCCCATGGATGAGGAATTGGTGACCTATTATCTTCAACAGAAGGTT
CTTGATAGCAGTTTTGCCTGCAGAGCCATTGCTGAAGTTGATCTGAACAAGTGTGAGC
CTTGGGATCTACCAGCCAGAGCCAAAATGGGAGAAAAGGAGTGGTACTTCTTCTCTCT
GAGGGACCGAAAGTATCCCACTGGTCTACGCACTAACAGAGCCACACAGGCAGGATA
CTGGAAAGCAACAGGGAAAGACAGAGAAGTTTTCAAAGGGAGAACTTCTATTCTTGT
GGGTATGAAGAAAACTCTGGTTTTCTACAAAGGGAGAGCACCCAAAGGGGAGAAAA
GCAACTGGGTAATGCATGAATACCGCCTGGAGGGCAAGTTTTCCTATTCCAATCTCCCC
AAGACCGCCAAGGATGAATGGGTGGTGACAAGAATATTTCAGAAATCTTGTGGACCA
AAGAAGGGCCCGCTAGCCCTGAGTAGGAGTTCAAATTATCTAAACGATTTGGACTCTC
CAGCTCTTCCTCCACTATTGGAATCGTCACCTTACGTCCCCTCAAATGGACAAGGAGG
CAGCAGCGAGTCAGTGGAACAGCATGTGACCTGCTTCTCCACTCCCATTGACAAATCT
TGCAGCAACAACTTCAACAGAGATCAATTCAGGAATCCTTCTGATACAGTAGACTACT
CTTTTCTCCACAATATCATGCAAAGTGACCCAGTTTACATGAACAACAATCCTAATGCC
TTAAGGCAGAGCTCCCATGATTTACATATAAATACCCCACAGCAGCTTCCATCATTTTC
CATGGCCCCAAATCCTCCAAACTTCCCAATGGTCTCTCAACTTTTCTCTCAAGGTGGTC
TTGGGCTTCCTTCAAATTCCCTTCTTAGGGCCCTCTATGAAGGAGTTGGCACCAATGAA
ATGATGGGCTTGAAGCAATGCAAGATGGAGCCACCCTCTTCCTCATTTGCTCATGACA
GAGTATGGGAAATGAATGCAAAATACAGATCAGTTAATAATTCTCAGCCTCAGCCTCA
GCCCATGGTCAGCATGTCACAAGAGACAGGACTCACATCAGAGATCAACAACACAG
AGATATCTAGTCAACTGACTCACTATCCAGAAGATCAGGGCAATCCTTCATCAGTTGA
CCTATCAGATTTCTGGTCTTCCTACTGA

ClNAC100 promoter sequence：

AAGCTTAGGTGAATGTATTGGAATATGTGTGTATAAGGATAAGGTGGGGGTCAAGTTTA
TGAGAATGTGTGTGTATAGGGGTGGGTAAAATCTAGAAAAATAAGCCCAACTTCATAC
AGTACTAGCACTTGCACCCATATGAGGTTCAAGGAATACGACATAGGAACAATCGACC
AATTTAGAAAATTTTCCGGCGTGAATTTTTTTACGGTGTGAGAGAATTCTAAGCACTAT
AATACTCACATATAAAAGCCTATCGACTAATTACACCATATATACATATATCAAGACATT
CTCTCAAAATAAACACAAAATTAAATAAAAAATGACATATGTATACAATATTATATTTA
ATTTTTTTTTTAAAAAAAAAACTCATTTTTCCCATTTATATTACTAAAGACAAAGAGAC
AAAGAGGTCTACTTTAATACAAATTGTGAAACATTTGTTGATAGAAAGAAAAAATAAT
AAAAATGCATTAACAACTTCAAAGTTTTCACTTCTTACCTCTTTATTTAAAACTATAAA
ACATAATTTATTTTTATATTTAAAACTTCTTTGTTGAGATAGATTTTTAAAAAACGATTTT
ATAATGTATGGCACAATAAAATGAGTTTAATAACAATTTAAATAATGCCTCCAAGAAAT
GGTCTTCGTATCAGAAAAATCTCCCTTTCTTCAATGGATGGTTTTTTTTTTAATTTTCAAA
ATTTTTTTGGGGGAAGTTAGGTAGACTAGTTAGCCGAAAGGGATTGTAACCTTCCACC
CTATAAGAAACGAGCATAAATAAATTGGACTGTTTCAACTCTTGCAGGGTAAGACCAT
GGAAAAGGGAGTACGTTTGACCTTAAACAGGGTTTGGTAGATGGCTCACATTTCTAGG
ACAGCGCGGGGGCGGAGGGCAATTTGGGAGACTGGGTCTGTGCGTTAAGGAAAATTT
CAAAAAATGAAGAAGTCGCTTCATAATATTAAATGCAAAAGTTTGTTTTCTCATGTGAC
CACGTGACTAACACTTTGCGGCGCTACAGTTTCTCTAGTCTTGTCGGGTTGCCAGCAG

GCGTGGAGTTTGTAGCCGGAAAAGATTTCCCGCTAATCGAGGCGCCGGCCAGACTTTA
TTACATGTCCTAACAGTGACGCCATATTTTCGTCAGTCATCAGCCGCGCAGTTAAATAG
GCAGCTTATCTCTTTTGCCGGGTAGAAATGACAAGTCAACAGTTAAATCATTTCAATTT
AAATAATAACAAGTCCCGTGAGTTTAAAACCGGGGCTTTGGCGCAACCCCCGCCGCG
GAAATTAACGACGTTAGGGTTAACCTGGGTTAGAGAGGCGGGGTTAGTAGAGGTCCCT
ACCACGTGGCGGCTTTGGGGACGTTGACTTGTTAGTGACGACGTGGAGCGTGGGGAG
CCGTGGATTTGAGGGCGCTGTCCCAGTTGGGCAATTTCGCGGGCAAAACGGGTGAAA
ATGAAAGAATTAGGAAAAGGTGAATGAAAGGAGGCCACGCGAAGGGAACGCGGGAT
TGGGAAGTTGTTACGTTGGGGGAGCTGACGTGGCTGGTGGAAAGCATGGGTGCATGG
TAATTGCTGCGCTGACGTGGCGGGGATACGTTGGGTTAGACCCCTTGAGAGGGGTGGA
AAACTGAAACAGGGTTAGGAAAGTTGGTTCGAGGAGAATCAGAAGTAGTTTTAGGTA
GATAGAGAGAGGGGGGGATGAGAAAGTTGCTTGCGGAGTACGTAATTCTGAGTCTTCT
GTCTCACTCTGCTGCACTTTCTCTATAAATCTAGGCTTGTTGTCTGAAGAGTTTTTAATA
ACAGCCTTAAGAGATAGAGCAGTATTATAGGAGTTAGAGGGAGGCCCCCAAACCCTG
AAACTGCCGCTTTTACTTGGTCTCTTTTATCTTTCTTTCTCTTTTAGCTTTTGAGAGCTGC
AGTGCAGTGCCTGACAGGTCTTTGTGTTAGCTTCTTGTTAAAGAAATCTGTGTCTATCA
GCACATTCTGAACATCATTCAATACATATACTACCCATTCACCATAGCTTCTTTTTTCTG
ACAACTGAACAGATACAGATACTGTTTGTTTGTCTGACTCTGTGCATGGGTGTATCATA
AACCCATTCTGCTTAATTCTGTCATTATTGGTGTCTGAGTCCTGTAAGTGTAAAGACTTT
GCTCGTGTGTGTAGTATAGA
